# A Multidisciplinary Model for the Governance of Clinical Innovation: Insights From a Qualitative Study of Australian Doctors

**DOI:** 10.1177/01632787251324662

**Published:** 2025-03-20

**Authors:** Miriam Wiersma, Ian Kerridge, Wendy Lipworth

**Affiliations:** 1 Sydney Health Ethics, 4334 The University of Sydney, NSW, Australia; 2 Haematology Department, Royal North Shore Hospital, Sydney, NSW, Australia; 3 Macquarie University Ethics and Agency Research Centre, 7788 Macquarie University, Sydney, NSW, Australia

**Keywords:** medical innovation, innovation ethics, innovation oversight, governance

## Abstract

Clinical innovation by doctors involves the development and use of interventions that have not been formally evaluated according to the usual standards of evidence-based medicine. While the distinction between research and innovation has been discussed theoretically, little is known about how doctors working in different specialty areas define and understand clinical innovation and how they distinguish it from other related practices. In order to address this gap, this qualitative interview study explored how doctors from diverse specialties defined and understood clinical innovation. Thirty-one semi-structured interviews were conducted with Australian doctors from surgery, reproductive medicine, and cancer care. While participants defined clinical innovation in similar ways, they also identified several morally and clinically salient characteristics that distinguish different types of innovation. Based on these findings, we developed a multidisciplinary governance model for clinical innovation that accounts for its diversity and complexity. This governance model offers clear guidance for determining what types of oversight are most appropriate for different types of clinical innovation. Its benefits include that it can be applied across diverse medical specialties and used alongside existing models, such as those used to identify clinical innovation.

## Introduction

Doctors play a vital role in developing novel medical interventions, including through “clinical innovation,” where they develop and use novel interventions that have not been formally evaluated and endorsed according to the usual standards of evidence-based medicine. Historical examples such as coronary artery bypass grafting and anesthesia demonstrate the transformative impact that clinical innovation can have on patient care (McKneally & Daar, 2003; Melly et al., 2018). At the same time, the birth defects resulting from the use of thalidomide for the treatment of nausea in pregnancy and failure of metal-on-metal hip replacements act as a reminder of the risks of clinical innovation (Hofbauer et al., 2013; Rehman et al., 2011).

Effective governance processes are critical for clinical innovation. Doctors need to recognize the benefits and risks of clinical innovation, and ensure their practice complies with governance processes (Samanta & Samanta, 2015). The oversight of clinical innovation, however, remains fragmented and challenging for doctors to navigate (Arts-De Jong et al., 2015; Davis Rebekah et al., 2022; Lloyd et al., 2022; Wiersma et al., 2023). Several factors complicate its effective governance.

First, the term “clinical innovation” lacks a consistent definition (Jiwa et al., 2009). It is often used interchangeably with terms such as “experimental treatment” and “new non-validated practice” (Grimmett & Sulmasy, 1998; Mastroleo & Holzer, 2020). Confusingly, it is also applied to the introduction of pilot studies and implementation of evidence-based treatments in health-care settings (Jiwa et al., 2009; O’Shea, 2012). A clear, operationalizable definition is, therefore, needed to facilitate the identification of clinical innovation and the different types of practices it includes in order to trigger appropriate oversight mechanisms (Agich, 2019; Birchley et al., 2019; Mastroleo & Holzer, 2020).

A second related challenge for the governance of clinical innovation is that it shares features of both clinical practice and research, each of which have different oversight and regulatory mechanisms (Wiersma et al., 2023). While stringent protocols protect the human rights of research participants through prospective ethical review and informed consent procedures (Czech et al., 2018; Shuster, 1997), the oversight of clinical practice is largely post hoc and takes place through professional standards, disciplinary procedures, and litigation (Samanta & Samanta, 2015). For oversight and regulatory purposes, clinical innovation is usually classified as a form of clinical practice (Wiersma et al., 2023). However, it differs in significant ways from standard clinical practice and in some ways resembles research more than practice (Wiersma et al., 2023).

A third challenge for the oversight of clinical innovation is that governance mechanisms are often developed for distinct medical specialties or specific innovative interventions (e.g., deep brain stimulation for depression in a pediatric population) (Davidson et al., 2018). This limits any recommendations made about definitional or oversight issues to that specific context. A holistic, empirically informed governance model for clinical innovation that can be generalized to different specialties and different innovative interventions (while also recognizing salient differences) is lacking (Wiersma et al., 2023).

These challenges have led to significant variation in how different types of innovative interventions are currently regulated (Wiersma et al., 2023). Many innovative interventions require no formal oversight—such as the use of “add-on” interventions (e.g., time-lapse imaging) in *in vitro* fertilization (Jones et al., 2021). Others are subject to more stringent oversight—such as the off-label use of ketamine for psychiatric conditions other than treatment-resistant depression, which in Australia can only be prescribed by psychiatrists with expertise in ketamine treatment and with informed consent from the patient (RANZCP, 2022).

To address these gaps and facilitate more nuanced and accessible governance of clinical innovation, this qualitative study explored how doctors from diverse medical specialties defined and understood clinical innovation, and its relationship to other practices (e.g., standard practice and research). This research builds on the limited and largely specialty-specific research that has been conducted to date, which suggests that the boundaries between clinical innovation, research and standard practice are blurred and difficult to determine (Agich, 2019; Birchley et al., 2019; King, 2003; Morreim et al., 2006), and that doctors have difficulty distinguishing innovation from practice variation (Elliot et al., 2019; Reitsma & Moreno, 2005; Rogers et al., 2014).

## Methods

Thirty-one participants were recruited between October 2020 and October 2021 using publicly available email addresses and snowball sampling. Potential research participants were identified and purposively recruited via Australian newspaper articles reporting on medical innovation, university newsletters announcing medical advancements, professional society websites (e.g., the Australian Medical Association website) and through targeted Google searches (e.g., “innovative surgeons New South Wales”). Invitations were sent to 163 individuals and participants were recruited from all Australian states. Doctors from three medical specialties—surgery, reproductive medicine, and cancer care (i.e., medical oncologists and hematologists) were approached. These medical specialties were chosen because they are known for their innovativeness and are diverse in terms of the nature of innovation within their specialties.

Interviews were conducted by MW (either by phone or Zoom), an experienced qualitative researcher, and lasted between 30 and 70 minutes. At the beginning of the interview, all participants were read a brief information statement about the project and its objectives, (Appendix A) which included the following statement explaining the researchers’ understanding of clinical innovation: clinical innovation is the development or use of novel interventions that differ from standard practice, and that have not yet been shown to be safe or effective according to the usual standards of evidence-based medicine.

Verbal or written consent was obtained from all participants. Participants were asked to explain their understanding of clinical innovation (i.e., to provide their own definition, whether or not it was consistent with the one underpinning the study) and describe their personal experience with innovative interventions. They were then asked about the drivers and deterrents of clinical innovation and their views about its oversight in Australia and whether it could be improved. The interview schedule was pilot tested with two experienced doctors and refined before use with participants.

Interviews were transcribed professionally or by MW and imported into NVIVO 12 (QSR International) for analysis. MW conducted all stages of the coding, with regular meetings with the broader team at which coding and interpretation were discussed. The coding procedure was informed by Charmaz’s outline of data analysis in grounded theory and Morse’s outline of the cognitive basis of qualitative research (Charmaz, 2006; Morse, 1994). This inductive approach involved an initial phase of focused line-by-line coding. These codes were then synthesized into broader descriptive categories, with the data subsequently re-analyzed to substantiate and enhance the categories and identify any new categories. Where appropriate, descriptive categories were then abstracted into high-level analytic concepts. The data analysis process was iterative and collaborative—with codes, categories and concepts refined and reorganized as new codes, categories and concepts were identified and discussed at regular team meetings. Data analysis continued until all categories were fully saturated (i.e., all codes fitted into one or more of the categories), and the key concepts were richly described and understood. This approach to qualitative data analysis has been utilized in other similar studies (Lipworth et al., 2015; Wiersma et al., 2020a).

Our research team then collaboratively developed a draft governance model, integrating our empirical findings with insights from existing literature, including two reviews that we have conducted (Wiersma et al., 2023; other work in preparation). We used a consensus-based approach to address discrepancies, enhancing the model’s comprehensiveness and applicability.

This study was approved by The University of Sydney Human Research Ethics Committee (protocol number 2019/868). The consolidated criteria for reporting qualitative studies (COREQ) checklist was used to guide the reporting of our results (Appendix B).

## Results

An overview of participants characteristics can be viewed in Table 1—most participants were male, located in New South Wales and had been in practice for over 10 years. Thematic saturation was reached after 12 interviews, and all 31 interviews were analyzed to ensure comprehensive data analysis and to capture any specialty specific nuances. A summary of key themes with illustrative quotes from our participants is shown in Table 2.

**Table 1:** Participants Characteristics

Characteristic	*n*	%
Specialty^a^		
Surgery	12	39
Cancer care	10	32
Reproductive medicine	7	23
Other^b^	2	6
Sex		
Female	5	16
Male	26	84
Years in practice		
10 years or less	8	26
20 years plus	7	23
30 years plus	14	45
Not disclosed	2	6
Australian state		
NSW	19	61
VIC	3	10
SA	4	13
WA	4	13
ACT	1	3

^a^Included seven pediatric specialists.

^b^Included one sports medicine physician and one endocrinologist who were interested in the use of stem cells as a form of clinical innovation.

**Table 2:** Key Themes and Illustrative Quotes

Theme	Key finding	Illustrative quote
Defining clinical innovation	Need for novelty and benefit	“The implication of “innovative” is that it works. You’re implying that it works. What if it killed the patient? (P21M Oncologist).”
		“So I think innovation implies that it’s a new and novel thing that shows benefit. Defining benefit is the vaguer area (P6M Surgeon).”
Dimensions of clinical innovation	Deliberate vs accidental innovation	“We have to jump through hoops to get things approved (P29F Hematologist).”
		“He did it because he made a hole in the oesophagus while he was operating, he repaired it and then reinforced it with a bit of stomach, he wrapped around it. What he found out afterwards was the patient’s reflux had gone (P7M Surgeon).”
	Major vs minor innovation	“So you have these creeps, things just gradually get better and occasionally have a jerk. Something comes out of the blue, there’s a major revolution in how you do things (P30M Fertility specialist).”
		“Trying different doses of known therapies, trying therapies for different indications (P9F Surgeon).”
		“Most people don’t do anything radically innovative, but they work around the boundaries of innovation (P23M, Fertility specialist).”
		“The whole world saw IVF processes sit on some pretty mega Australian innovations (P31M Fertility specialist).”
	Simplifying vs complexifying innovation	“New is always attractive, but “the new” tends to be technology-based new, always. And that’s the way the world is. And we kind of follow that (P10M Surgeon).”
		“One of the greatest innovations we’ve had…was to treat Achilles tendon tears without surgery. That’s been a great innovation, but nobody sees that as an innovation, they see that as a retrograde step (P10M Surgeon).”
	Necessary vs voluntary innovation	“I just loved it, for the technical aspects, always been tinkering with things (P1M Surgeon).”
		“When paediatric catheters suddenly went out of stock, “we all came up with our own little solutions” (P15M Surgeon).”
		“It’s forced innovation and it’s definitely not for improving patient care. It’s basically to plug holes in patient care (P15M Surgeon).”
Relationship to other practices	Standard practice	“Clinical innovation is a vital part of standard practice which ensures that you’ll be better next year than you were this year. It should be seen as part of routine care in a way (P20M Oncologist).”
		“You have a patient in front of you who’s desperate for something and there’s some evidence in a different circumstance…In clinical trials you’re doing the same, but you’re doing it in a structured way (P27M Oncologist).”
	Compassionate access and other special access schemes	“They have “a level of safety or preliminary clinical efficacy” and they are often “registered and approved for use” in other countries (P12M Surgeon).”
	Off-label use	“Off-label use of drugs, well that’s just part of the course (P4M Surgeon).”
		“Due to its high cost, “companies can’t be bothered updating the label” (P32M Oncologist).”
		“I guess it’s a degree of innovation (P32M Oncologist).”
		“50% of all drugs used in oncology…are used outside their approval (P21M Oncologist).”

### Defining and Classifying Clinical Innovation

#### Need for Novelty and Benefit

Novelty and benefit were described as key defining features of clinical innovation by doctors from all specialties. “Novelty” included the use of new medical devices and pharmaceuticals or using existing devices and pharmaceuticals in new ways—for example, prescribing a cancer drug for an indication it was not approved for. Some doctors were clear that for an intervention to be considered innovative, novelty alone was insufficient—it had to be a new idea that worked and benefited patients. Here, several doctors acknowledged the difficulty of defining “benefit”, with cancer physicians noting that benefit was not necessarily about “cure” or improving overall survival rates, but also improving patients’ quality of life or convenience (e.g., requiring treatment monthly vs. fortnightly). Other doctors rejected the notion of innovation as novelty, citing the difficulty of defining “new.” These doctors highlighted the value-laden nature of terms like “innovation” and “new,” claiming that these terms could perpetuate a problematic “newer is better” (P10M: Surgeon) mentality in practice.

### Dimensions of Clinical Innovation

Participants not only provided definitions of innovation but also described it in ways that revealed four key dimensions. These dimensions serve as important distinguishing characteristics of clinical innovation.

#### Deliberate Versus Accidental Innovation

Doctors noted that while many innovations were deliberately developed, others emerged accidentally and later proved successful. One notable example of the latter was intracytoplasmic sperm injection for the treatment of male factor infertility. Here doctors’ original intent had been to inject the sperm just under the egg’s shell, using a technique known as SUZI—subzonal sperm injection. However, they had accidentally injected it further—into the egg itself—and achieved a successful pregnancy. This case exemplifies accidental innovation’s key features: it was unintentional (i.e., the doctors had not purposively attempted to solve a specific clinical problem) and it had an unexpected positive outcome (i.e., a significant clinical problem was solved).

#### Major Versus Minor Innovation

Doctors were cognizant that while clinical innovation is often associated with major breakthroughs by patients and doctors alike, most innovation involves gradual refinements that take place over years. Numerous examples of incremental innovation were discussed, while truly transformative innovation was perceived to be rare. One exception to this was provided by participants working in assisted reproductive technology (ART), who provided numerous examples of transformative innovations, with several noting that the ART industry in Australia was known for capacity to generate such innovations.

#### Simplifying Versus Complexifying Innovation

Participants sometimes distinguished between innovations that added complexity to healthcare and those that simplified treatments for patients. Many doctors described the appeal of technologically sophisticated innovations for patients, doctors and the media. At the same time, the importance of low-cost, low-tech innovations was emphasized, as was their potential to be of greater benefit for a broader range of patients, including those from developing nations with limited resources. This included innovations that could simplify care, such as doing procedures under local versus general anesthetic in the surgical setting.

#### Necessary Versus Voluntary Innovation

While participants frequently described innovation that they had willingly and sometimes excitedly been involved in, they noted that innovation was also born out of necessity in certain situations. Several surgeons described how they had been forced to innovate when there were device shortages. Necessary innovation was seen to be particularly prevalent in the pediatric context where a lack of specialized (and sufficiently small) devices meant that surgeons had create their own solutions. Additionally, as drugs were typically approved for adult, not pediatric patients, doctors frequently had to use drugs off-label or access them through compassionate access schemes. Necessary innovation was viewed with some skepticism, with several doctors commenting that it was not about improving patient care, but rather about closing gaps in treatment.

### Relationship to Other Practices

Doctors spoke of the relationship between clinical innovation and other practices—including standard practice, clinical research, compassionate and special access schemes, and off-label prescribing.

#### Relationship to Standard Practice

For the most part, doctors described clinical innovation as distinct from standard practice. While standard practice included treatments doctors used on a regular basis, innovations were seen to be those that were either completely different to existing treatment modalities or that pushed at the limits of the medical system. One exception to this arose in interviews with medical oncologists, with several participants claiming that clinical innovation was part of everyday practice and an integral way of improving patient care.

#### Relationship to Research

Some medical oncologists equated clinical innovation with research, stating that it included offering patients’ novel treatments in clinical trials. These participants also asserted that clinical innovation included the use of the outputs of standard research processes. For example, when asked to describe “successful” or “unsuccessful” clinical innovations, several oncologists discussed monoclonal antibodies, CAR-T cells, and the use of molecular data to inform cancer treatment. Even where research was not seen as a form of clinical innovation, the close relationship between the two was noted, with knowledge flowing both “from the bench to the clinic, and clinic to the bench” (P21M: Oncologist).

#### Relationship to Compassionate Access and Other Special Access Schemes

Accessing and using medicines through special access schemes were frequently described as a form of clinical innovation by doctors across all specialties. In Australia, these schemes include the Special Access Scheme administered by the Australian Therapeutic Goods Administration and compassionate access programs run by pharmaceutical companies. Innovative interventions accessed via these schemes were perceived to have a level of evidence supporting their use.

Pharmaceutical companies were seen to be key players in facilitating or inhibiting clinical innovation as they could approve or deny doctors’ requests for access to innovative medicines through compassionate access programs, which were particularly common in the setting of rare diseases and pediatrics. These schemes were perceived to have multiple benefits—including benefiting patients and prompting formal research. There were, however, perceived drawbacks, with doctors expressing frustration that they had to “push for compassionate access” (P29F: Oncologist) from pharmaceutical companies or that patients could lose access to an intervention if a scheme was discontinued.

#### Relationship to Off-Label Prescribing

Off-label prescribing (i.e., the use of interventions approved by a regulator outside the parameters of approval) was frequently raised as an example of clinical innovation. Doctors discussed reasons for off-label prescribing, attributing it partly to pharmaceutical companies’ reluctance to pursue formal approval for novel indications to due to the prohibitive costs involved. These participants explained that while regulators may approve medicines for one specific indication, it was not uncommon for them to have multiple, well-established evidence-based uses. Pediatrics was again raised as an example where most medication used was off-label, due to the lack of clinical trials and medicines approved for pediatric populations.

Drawbacks to off-label prescribing were noted, including that doctors sometimes had to go through formal processes to use medicines off-label, including for example, via an application to the hospital drug committee. Some doctors expressed concern that data (including patient-follow up information) was not collected when medicines were used off-label. In order to protect patients who received off-label interventions, several doctors highlighted the importance of gaining informed consent. Due to the frequency in which it occurs, however, several doctors questioned whether off-label prescribing was indeed “innovative.” These doctors were reluctant to consider off-label prescribing as a form of innovation because it was so widespread throughout medicine—with one surgeon giving the example of the frequent off-label use of Mirabegron to treat bladder problems in children (P16M: Surgeon).

## Discussion

### Summary of Results

This interview study of thirty-one Australian doctors from diverse specialties—including surgery, reproductive medicine, and cancer care—reveals that participants defined clinical innovation similarly, while identifying distinct dimensions of the practice. For the most part, our participants clearly distinguished clinical innovation from practice and research, although there was a notable exception to this within the group of oncologists, some of whom described clinical innovation as being part of standard practice and/or research. Participants also recognized several practices that are examples of, or that facilitate clinical innovation, including off-label prescribing, and special/compassionate access schemes.

### Conceptual and Practical Implications

#### Defining Innovation

Prior empirical studies, primarily focused on surgeons, found that doctors struggled to define clinical innovation (Apramian et al., 2015; Zahra et al., 2020), often defining it broadly as the development of health services (Day-Duro et al., 2020; Esfahani et al., 2022), or as a way of responding to patients’ needs (Elliot et al., 2019). In their qualitative interview study with surgeons, Rogers et al. (2014) found that surgeons view innovation as having five key defining features: newness, degree of change, level of risk, impact and requirement for formal processes. Our participants highlighted two of these features: novelty and benefit (described in Roger’s study as impact) (Rogers et al., 2014). Unlike prior empirical studies, however (Apramian et al., 2016; Esfahani et al., 2022; Rogers et al., 2014), a small subset of our participants were skeptical of definitions that equated clinical innovation with newness. Our participants also emphasized the complexities of defining benefit for innovative interventions. This reflects discussions in the theoretical literature on the problematic nature of the term innovation because it is a heavily value-laden concept that has positive connotations that might not always be warranted (Birchley et al., 2019; Mastroleo & Holzer, 2020).

Given these complexities, defining clinical innovation based on its novelty and benefit may be problematic, particularly due to the inherent subjectivity involved in making these evaluations and the fact that benefit can only be established retrospectively. The definition used in our study (the development and use novel interventions that have not been formally evaluated and endorsed according to the usual standards of evidence-based medicine) accommodates these complexities, because it does not rely on benefit as a defining characteristic of innovation, but rather on evidence. We suggest retaining the use of the term “novel” despite definitional challenges, as the use of non-evidence-based interventions that are well established cannot meaningfully be considered to be a form of innovation.

#### Distinguishing Between Innovation, Research, and Standard Practice

Other studies have shown that the boundaries between clinical innovation, research and standard practice are blurred and are not always easy to determine (Agich, 2019; Birchley et al., 2019; King, 2003; Morreim et al., 2006). Studies have also highlighted the difficulties doctors experience distinguishing innovation from practice variation (Apramian et al., 2016; Elliot et al., 2019; Reitsma & Moreno, 2005; Rogers et al., 2014). While our participants did not focus on innovation versus practice variation, they did reflect on the distinction between innovation, research, and standard practice. Most viewed clinical innovation as distinct from research and standard practice, but as in previous studies (Reitsma & Moreno, 2005; Rogers et al., 2014), they did not offer clear demarcations. Furthermore, some medical oncologists rejected our proposed definition of clinical innovation, describing it as part of standard practice or as synonymous with research participation. While the slippage between the three activities for these participants could be due to them having a profoundly different definition of innovation, it also potentially could be due to the collapsing boundaries between research, innovation and standard practice in cancer care (Heynemann et al., 2023). As our participants explained, treating cancer patients frequently involved enrolment in clinical trials, which they perceived to be both innovative and part of their everyday practice.

This suggests that there is merit in retaining the essential meaning of clinical innovation as distinct from both research and care, while recognizing that *in practice* a particular activity might fall into more than one category. For this reason, it could be worthwhile adding to the definition used in this study: novel interventions *used outside the research context* that differ from standard practice, and that have not yet been shown to be safe or effective according to the usual standards of evidence-based medicine.

#### Governing Clinical Innovation

Various frameworks guide the oversight of clinical innovation (Wiersma et al., 2023). Many, however, address innovation in medicine broadly, rather than focusing specifically on clinical innovation (Fleuren et al., 2004; Lloyd et al., 2022). Fleuren’s theoretical framework, for example, which outlines the main stages of the innovation process and its determinants, classifies health promotion strategies and electronic medical record systems as clinical innovation (Fleuren et al., 2004). Other frameworks focus narrowly on specific domains—most typically surgery (Wiersma et al., 2023). The IDEAL framework, for example, describes the stages of surgical innovation and proposes a structure for evaluating the ethical issues at each stage (Barkun et al., 2009; Wiersma et al., 2023). While its Stage 1 or “Idea” stage is akin to clinical innovation, IDEAL primarily addresses surgical innovations that follow a sequential process from proof of concept through to evaluation in clinical research (Barkun et al., 2009; Wiersma et al., 2023).

### A Governance Model for Clinical Innovation

Broadly speaking, there are six levels of oversight of clinical innovation (Gupta et al., 2018; Karpowicz et al., 2016). Individual level oversight is based on professional codes of conduct and standards of practice. Peer oversight includes both informal and formal review by colleagues or mentors. Departmental oversight includes formal and informal review by chief of the department, case reviews, and morbidity and mortality conferences. Institutional oversight includes review by institutional ethics committees or innovation committees (where available), quality control measures and audits. Professional oversight includes clinical practice guidelines and policies from medical societies or professional associations. Governmental oversight includes state and federal policies, guidelines and regulations. This includes those from the health department and relevant regulatory agencies (e.g., the Therapeutic Goods Administration in Australia). It also includes health complaint bodies, consumer protection laws, criminal and civil legal regimes (Gupta et al., 2018; Karpowicz et al., 2016). At each of these levels, governance mechanisms can range from less strict and restrictive mechanisms, such as individual decision making and peer review, through to more stringent mechanisms such as laws. For example, departmental oversight may include both informal review by peers or managers, or formal procedures, such as written requests to use an innovative intervention to the relevant departmental committee.

**Figure 1:**
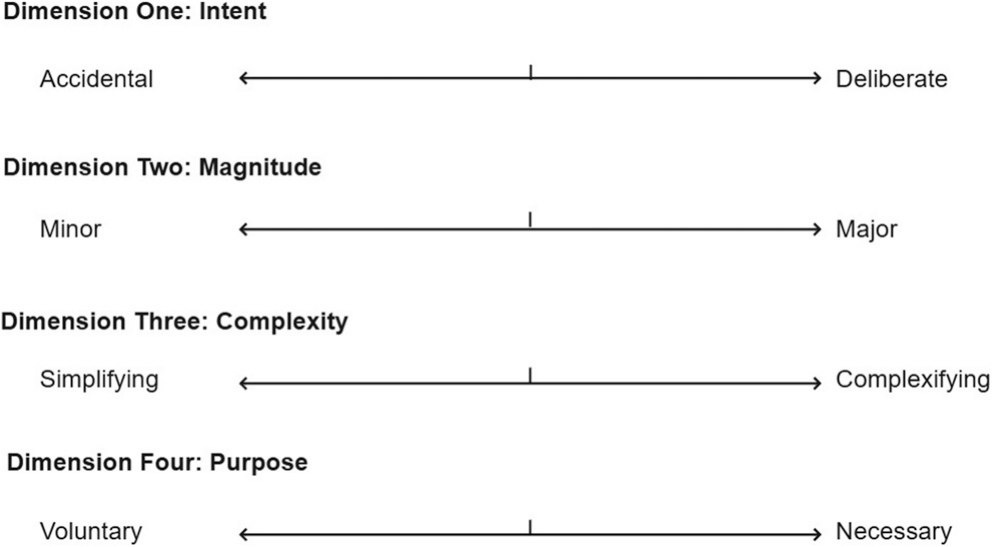
A Governance Model for Clinical Innovation

Based on the results of this study, we suggest that at each of these levels there are four dimensions of clinical innovation that can help to determine what level of oversight is necessary, and what specific form this oversight should take. Each dimension portrays two opposing characteristics of clinical innovation and the spectrum between the two in which an innovative intervention may lie, as shown in Figure 1. For example, an innovative intervention may lie anywhere between minor versus major innovation on *Dimension 2 – Magnitude*. Below we describe the four dimensions of clinical innovation and their characteristics.

#### Dimension 1—Intent

Accidental innovation is an unintentionally developed solution to a specific clinical problem that can only be identified retrospectively. For example, intracytoplasmic sperm injection. Deliberate innovation is deliberately undertaken to solve a specific patient problem. It involves pre-planning, formal approval and patient consent, for example, the use of compassionate access or special access schemes.

#### Dimension 2—Magnitude

Minor innovations include small changes to clinical practice that may be difficult to distinguish from routine variation. For example, moving from cardiotocography-based fetal monitoring to wireless electrode monitoring. Major innovations are transformative changes to clinical practice that may disrupt existing patient care pathways, require additional resources for its implementation, and necessitate additional training by doctors. For example, repurposing a drug for a completely different indication and population.

#### Dimension 3—Complexity

Simplifying innovation involves streamlining treatment to benefit patients in some way and is often driven by resource constraints. For example, knee arthroscopy under general local anesthetic and minimal ovarian stimulation protocols in In vitro fertilization. Complexifying innovation complicates the patient care pathway in some way— including by requiring additional resources, training of personnel or specialized equipment. For example, the Da Vinci Robot.

#### Dimension 4—Purpose

Voluntary innovation is driven by a doctor’s interest in solving a specific patient problem or using a novel technique. For example, early adoption of intracytoplasmic sperm injection by fertility clinicians. Necessary innovation is used to address gaps in patient care or provide treatment when there are no alternatives. For example, pediatricians’ modification of adult devices for pediatric surgery.

This model illustrates the diversity of clinical innovation and the relationships and tensions between its different types. It is also designed to inform the oversight of clinical innovation—with oversight based on where a specific intervention lies on each of the four dimensions. For example, accidental innovation can only have retrospective oversight, such as review through morbidity and mortality conferences, quality control measures, or audit and feedback processes. This model also indirectly accounts for risk as the four dimensions capture different elements of risk—with interventions that lie at the accidental, major and complexifying sides of the scale more likely to be high risk.

The above case studies demonstrate how the model could be used to inform the oversight of clinical innovation based on its characteristics (see Table 3). It is arguable that had this model been available and used in the past, it might have prevented harms associated with the use of transvaginal mesh (TVM) for pelvic organ prolapse, which harmed hundreds of thousands of women worldwide when introduced without formal oversight in the late 1990s (Wiersma et al., 2020b). Within our model, TVM would have fallen at the deliberate, major, complexifying and necessary sides of the four dimensions—which points to the need for comprehensive formal oversight including informed consent, written approval by chief of surgery, and registration in a national registry.

**Table 3:** Clinical Innovation Case Studies

Innovative intervention	Ketamine for treatment resistant depression	Titanium elastic nails for complex pelvic fractures	Mild ovarian stimulation in IVF
Overview	Ketamine was originally approved as an anesthetic in the 1970s (Segal & Sisti, 2016). It has been used off-label since the 1980s for the treatment of psychiatric disorders, including treatment-resistant depression (Segal & Sisti, 2016). It has come under scrutiny due to the lack of oversight, and concerns that patients are not being fully informed about its “experimental” nature (Wilkinson & Sanacora, 2017).	Complex pelvic fractures caused by major traumas (e.g., car accidents) are sometimes treated using minimally invasive techniques with percutaneous screw fixation. A 2024 retrospective study reports on the use of minimally invasive techniques using titanium elastic nails to repair complex pelvic fractures in 24 patients (Jia et al., 2024). Titanium elastic nails were originally indicated for use in lower and upper extremity fractures.	Mild ovarian stimulation protocols: where a lower (<150 IU) dose of gonadotropins is given with the intention of limiting the number of oocytes (Nargund et al., 2022). Emerging evidence suggests that mild stimulation is less costly, safer, and as effective as conventional IVF protocols. There is continuing debate within the reproductive medicine community about its effectiveness, despite several RCTs and reviews.
Dimensions of clinical innovation	Dimension 1: Deliberate Dimension 2: Middle (neither minor/major) Dimension 3: Middle (neither simplifying or complexifying) Dimension 4: Voluntary	Dimension 1: Deliberate Dimension 2: Major (novel device used, patient care pathway changed) Dimension 3: Complexifying Dimension 4: Voluntary	Dimension 1: Deliberate Dimension 2: Minor Dimension 3: Simplifying Dimension 4: Voluntary
Oversight mechanisms	Because it is a deliberate and voluntary innovation, there should be scope for clinician and patient autonomy. However, because ketamine is a dangerous and legally controlled substance, there is a need for a higher level of oversight, including peer review, and/or departmental and government regulation to ensure that the substance is not inappropriately prescribed or misused.	Because this is a major innovation with potential for harm, governance is required at all levels and is justifiably stringent. For example, at the departmental level, there could be a requirement for written approval by chief of surgery. At a government level, there could be a requirement for a written request to regulator and registration in national registry.	Because this is a minor procedure that poses little risk, peer review may be sufficient to ensure that it is not over-used and that it does not have unexpected adverse effects that are only evident when larger groups are observed.

This model both has broad applicability across medicine and is flexible, describing means of oversight that may be tailored to the unique characteristics of specific interventions. Importantly it demonstrates that the oversight of clinical innovation may be more meaningfully discussed in terms of its characteristics, rather than by the medical specialty in which it occurs. This model could be integrated with, or used alongside other models, such as those designed to identify clinical innovation and distinguish it from practice variation (Hutchison et al., 2015).

### Strengths and Limitations

This is the first empirical study to compare and analyze how doctors from diverse specialties define, identify, and understand clinical innovation. Its large sample size for qualitative research allowed for in-depth exploration of doctors’ experiences with and attitudes towards clinical innovation. This rich data enabled the development of a broadly applicable governance model for clinical innovation.

However, limitations exist. Given that our participants were senior and highly experienced in the use of innovative interventions, their views may not be representative of their specialty. The use of purposive and snowballing techniques, while providing rich data, limits generalizability to medical professionals who have experience or interest in innovative interventions. Furthermore, our participants’ views may not reflect those of medical professionals in diverse global health systems. Further research is needed to test the model across different contexts and medical specialties.

## Conclusion

This study provides an empirically informed definition of clinical innovation and a governance model that accounts for both the complexity and diversity of innovative interventions across different medical specialties. It offers key clinical innovation stakeholders (e.g., clinicians, institutions, and policy makers) a clear way of classifying innovative interventions across four dimensions in order to determine whether oversight is necessary, and if so, what types of oversight mechanisms are most appropriate.

## Supplemental Material

Supplemental Material - A Multidisciplinary Model for the Governance of Clinical Innovation: Insights From a Qualitative Study of Australian Doctors Supplemental Material for A Multidisciplinary Model for the Governance of Clinical Innovation: Insights From a Qualitative Study of Australian Doctors by Miriam Wiersma, Ian Kerridge, and Wendy Lipworth in Evaluation & the Health Professions

Supplemental Material - A Multidisciplinary Model for the Governance of Clinical Innovation: Insights From a Qualitative Study of Australian Doctors Supplemental Material for A Multidisciplinary Model for the Governance of Clinical Innovation: Insights From a Qualitative Study of Australian Doctors by Miriam Wiersma, Ian Kerridge, and Wendy Lipworth in Evaluation & the Health Professions

## References

[bibr1-01632787251324662] Agich G. J. (2019). Knowing one’s way around: The challenge of identifying and overseeing innovations in patient care. The American Journal of Bioethics, 19(6), 1–3. 10.1080/15265161.2019.1611275 31135313

[bibr2-01632787251324662] Apramian T. Cristancho S. Watling C. Ott M. Lingard L. (2016). “They have to adapt to learn”: Surgeons’ perspectives on the role of procedural variation in surgical education. Journal of Surgical Education, 73(2), 339–347. 10.1016/j.jsurg.2015.10.016 26705062 PMC5578763

[bibr3-01632787251324662] Apramian T. Watling C. Lingard L. Cristancho S. (2015). Adaptation and innovation: A grounded theory study of procedural variation in the academic surgical workplace. Journal of Evaluation in Clinical Practice, 21(5), 911–918. 10.1111/jep.12398 26096874 PMC5578747

[bibr4-01632787251324662] Arts-De Jong M. Harmsen M. G. Hoogerbrugge N. Massuger L. F. Hermens R. P. De Hullu J. A. (2015). Risk-reducing salpingectomy with delayed oophorectomy in BRCA1/2 mutation carriers: Patients’ and professionals’ perspectives. Gynecologic Oncology, 136(2), 305–310. 10.1016/j.ygyno.2014.12.031 25560807

[bibr5-01632787251324662] Barkun J. S. Aronson J. K. Feldman L. S. Maddern G. J. Strasberg S. M. Balliol Collaboration Altman D. G. Barkun J. S. Blazeby J. M. Boutron I. C. Campbell W. B. Clavien P. A. Cook J. A. Ergina P. L. Flum D. R. Glasziou P. Marshall J. C. McCulloch P. Nicholl J. Vandenbroucke J. (2009). Evaluation and stages of surgical innovations. Lancet, 374(9695), 1089–1096. 10.1016/S0140-6736(09)61083-7 19782874

[bibr6-01632787251324662] Birchley G. Ives J. Huxtable R. Blazeby J. (2019). Conceptualising surgical innovation: An eliminativist proposal. Health Care Analysis, 28(1), 73–97. 10.1007/s10728-019-00380-y PMC7045746 31327091

[bibr7-01632787251324662] Charmaz K. (2006). Constructing grounded theory: A practical guide through qualitative analysis. Sage Publications.

[bibr8-01632787251324662] Czech H. Druml C. Weindling P. (2018). Medical ethics in the 70 years after the Nuremberg Code, 1947 to the present. Wiener Klinische Wochenschrift, 130(Suppl 3), 159–253. 10.1007/s00508-018-1343-y 29926188

[bibr9-01632787251324662] Davidson B. Elkaim L. Lipsman N. Ibrahim G. (2018). Editorial. An ethical framework for deep brain stimulation in children. Neurosurgical Focus, 45(3), Article E11. 10.3171/2018.7.FOCUS18219 30173615

[bibr10-01632787251324662] Davis Rebekah A. Lee Kachiu C. Lee Ivy A. Levin Yakir S. Garibyan L. (2022). Innovating on innovation training with the Virtual Magic Wand (VMW) program: A qualitative study. Archives of Dermatological Research, 315(3), 513–519. 10.1007/s00403-022-02392-6 36121556 PMC9483859

[bibr11-01632787251324662] Day-Duro E. Lubitsh G. Smith G. (2020). Understanding and investing in healthcare innovation and collaboration. Journal of Health Organization and Management, 34(4), 469–487. 10.1108/JHOM-07-2019-0206 32250574

[bibr12-01632787251324662] Elliot T. Miola J. Samanta A. Samanta J. (2019). Fears and fallacies: Doctors’ perceptions of the barriers to medical innovation. Clinical Ethics, 14(4), 155–164. 10.1177/1477750919886090

[bibr39-01632787251324662] Esfahani M. Heydari Khajehpour S. Roushan-Easton G. Howell R. (2022). A framework for successful adoption of surgical innovation. Surgical Innovation, 29(5), 662–670. 10.1177/15533506221074612 35315708 PMC9615345

[bibr13-01632787251324662] Fleuren M. Wiefferink K. Paulussen T. (2004). Determinants of innovation within health care organizations: Literature review and Delphi study. International Journal for Quality in Health Care, 16(2), 107–123. 10.1093/intqhc/mzh030 15051705

[bibr14-01632787251324662] Grimmett M. Sulmasy D. (1998). The call of the sirens: Ethically navigating the sea of nonvalidated therapies. Journal of Refractive Surgery, 14(5), 559–566. 10.3928/1081-597X-19980901-15 9791823

[bibr15-01632787251324662] Gupta S. Muskens I. S. Fandino L. B. Hulsbergen A. F. C. Broekman M. L. D. (2018). Oversight in surgical innovation: A response to ethical challenges. World Journal of Surgery, 42(9), 2773–2780. 10.1007/s00268-018-4565-2 29536142 PMC6097786

[bibr16-01632787251324662] Heynemann S. Lipworth W. McLachlan S. A. Philip J. John T. Kerridge I. (2023). When research becomes practice: The concept of the therapeutic misconception and challenges to consent in clinical trials. Internal Medicine Journal, 53(2), 271–274. 10.1111/imj.16015 36822606

[bibr17-01632787251324662] Hofbauer M. Muller B. Murawski C. D. Karlsson J. Fu F. H. (2013). Innovation in orthopaedic surgery as it relates to evidence-based practice. Knee Surgery, Sports Traumatology, Arthroscopy, 21(3), 511–514. 10.1007/s00167-012-2360-4 23287893

[bibr18-01632787251324662] Hutchison K. Rogers W. Eyers A. Lotz M. (2015). Getting clearer about surgical innovation: A new definition and a new tool to support responsible practice. Annals of Surgery, 262(6), 949–954. 10.1097/SLA.0000000000001174 25719812

[bibr19-01632787251324662] Jia Z. Qin H. Lin J. Wang X. Bai R. Zou S. Huang W. Hu X. (2024). Minimally invasive treatment of pelvic fractures with titanium elastic nailing: An innovative technology. Surgical Innovation, 31(4), 373–380. 10.1177/15533506241249260 38654530

[bibr20-01632787251324662] Jiwa M. McKinley R. K. Spilsbury K. Arnet H. Smith M. (2009). Deploying a clinical innovation in the context of actor-patient consultations in general practice: A prelude to a formal clinical trial. BMC Medical Research Methodology, 9, Article 54. 10.1186/1471-2288-9-54 19615058 PMC2716367

[bibr21-01632787251324662] Jones G. L. Lang V. Hudson N. (2021). A baby at all costs? Exploring the use and provision of unproven adjuvant treatments in the context of IVF. Seminars in Reproductive Medicine, 39(5-6), 220–226. 10.1055/s-0041-1731789 34500475

[bibr22-01632787251324662] Karpowicz L. Bell E. Racine E. (2016). Ethics oversight mechanisms for surgical innovation: A systematic and comparative review of arguments. Journal of Empirical Research on Human Research Ethics, 11(2), 135–164. 10.1177/1556264616650117 27329472

[bibr23-01632787251324662] King N. (2003). The line between clinical innovation and human experimentation. Seton Hall Law Review, 32(3), 571–580. https://scholarship.shu.edu/shlr/vol32/iss3/5/12741413

[bibr24-01632787251324662] Lipworth W. Kerridge I. Morrell B. Forsyth R. Jordens C. F. (2015). Views of health journalists, industry employees and news consumers about disclosure and regulation of industry-journalist relationships: An empirical ethical study. Journal of Medical Ethics, 41(3), 252–257. 10.1136/medethics-2013-101790 24603036

[bibr25-01632787251324662] Lloyd S. FitzGerald G. Collie J. Cliff C. (2022). What ‘sparks’ innovation in rural health settings: A case study. Asia Pacific Journal of Health Management, 17(3), 14–31. 10.24083/apjhm.v17i3.1609

[bibr26-01632787251324662] Mastroleo I. Holzer F. (2020). New non-validated practice: An enhanced definition of innovative practice for medicine. Law, Innovation and Technology, 12(2), 318–346. 10.1080/17579961.2020.1815405

[bibr27-01632787251324662] McKneally M. Daar A. (2003). Introducing new technologies: Protecting subjects of surgical innovation and research. World Journal of Surgery, 27(8), 930–935. 10.1007/s00268-003-7096-3 12822049

[bibr28-01632787251324662] Melly L. Torregrossa G. Lee T. Jansens J. L. Puskas J. D. (2018). Fifty years of coronary artery bypass grafting. Journal of Thoracic Disease, 10(3), 1960–1967. 10.21037/jtd.2018.02.43 29707352 PMC5906252

[bibr29-01632787251324662] Morreim H. Mack M. J. Sade R. M. (2006). Surgical innovation: Too risky to remain unregulated? The Annals of Thoracic Surgery, 82(6), 1957–1965. 10.1016/j.athoracsur.2006.07.003 17133664

[bibr30-01632787251324662] Morse J. M. (1994). Emerging from the data: The cognitive processes of analysis in qualitative inquiry. In Morse J. M. (Ed.), Critical issues in qualitative research methods (pp. 23–43). Sage Publications.

[bibr31-01632787251324662] Nargund G. Datta A. K. Campbell S. Patrizio P. Chian R.-C. Ombelet W. Von Wolff M. Lindenberg S. Frydman R. Fauser B. C. (2022). The case for mild stimulation for IVF: Recommendations from The International Society for Mild Approaches in Assisted Reproduction. Reproductive Biomedicine Online, 45(6), 1133–1144. 10.1016/j.rbmo.2022.07.019 36220713

[bibr32-01632787251324662] O’Shea K. (2012). Infusion management: One journey in clinical innovation. Biomedical Instrumentation & Technology, 46(1), 25–28. 10.2345/0899-8205-46.1.25 22239353

[bibr33-01632787251324662] RANZCP . (2022). Clinical memorandum: Use of ketamine in psychiatric practice. https://www.ranzcp.org/getmedia/75baa529-2b71-419f-993a-2ff64ede50fe/cm-use-ofketamine-in-psychiatric-practice.pdf

[bibr34-01632787251324662] Rehman W. Arfons L. M. Lazarus H. M. (2011). The rise, fall and subsequent triumph of thalidomide: Lessons learned in drug development. Therapeutic Advances in Hematology, 2(5), 291–308. 10.1177/2040620711413165 23556097 PMC3573415

[bibr35-01632787251324662] Reitsma A. M. Moreno J. D. (2005). Ethics of innovative surgery: US surgeons’ definitions, knowledge, and attitudes. Journal of the American College of Surgeons, 200(1), 103–110. 10.1016/j.jamcollsurg.2004.09.032 15631926

[bibr36-01632787251324662] Rogers W. A. Lotz M. Hutchison K. Pourmoslemi A. Eyers A. (2014). Identifying surgical innovation: A qualitative study of surgeons’ views. Annals of Surgery, 259(2), 273–278. 10.1097/SLA.0b013e31829ccc5f 23787218

[bibr37-01632787251324662] Samanta J. Samanta A. (2015). Quackery or quality: The ethicolegal basis for a legislative framework for medical innovation. Journal of Medical Ethics, 41(6), 474–477. 10.1136/medethics-2014-102366 25552664

[bibr38-01632787251324662] Segal A. Sisti D. (2016). Research moratoria and off-label use of ketamine. The American Journal of Bioethics, 16(4), 60–61. 10.1080/15265161.2016.1145285 26982929

[bibr40-01632787251324662] Shuster E. (1997). Fifty years later: The significance of the Nuremberg Code. New England Journal of Medicine, 337(20), 1436–1440. 10.1056/NEJM199711133372006 9358142

[bibr41-01632787251324662] Wiersma M. Kerridge I. Lipworth W. (2020a). Status, respect and stigma: A qualitative study of non-financial interests in medicine. Journal of Bioethical Inquiry, 17(2), 203–216. 10.1007/s11673-020-09970-1 32162158

[bibr42-01632787251324662] Wiersma M. Kerridge I. Lipworth W. (2020b). Transvaginal mesh, gender and the ethics of clinical innovation. Internal Medicine Journal, 50(5), 523–526. 10.1111/imj.14833 32431042

[bibr43-01632787251324662] Wiersma M. Kerridge I. Lipworth W. (2023). Clinical innovation ethics frameworks: A systematic narrative review. Health Policy, 129, Article 104706. 10.1016/j.healthpol.2023.104706 36639310

[bibr44-01632787251324662] Wilkinson S. Sanacora G. (2017). Considerations on the off-label use of ketamine as a treatment for mood disorders. JAMA, 318(9), 793–794. 10.1001/jama.2017.10697 28806440 PMC6248331

[bibr45-01632787251324662] Zahra J. Paramasivan S. Blencowe N. S. Cousins S. Avery K. Mathews J. Main B. G. McNair A. G. K. Hinchliffe R. Blazeby J. M. Elliott D. (2020). Discussing surgical innovation with patients: A qualitative study of surgeons’ and governance representatives’ views. BMJ Open, 10(11), Article e035251. 10.1136/bmjopen-2019-035251 PMC7651722 33158818

