# Supplementary material for: A Multidisciplinary Model for the Governance of Clinical Innovation: Insights From a Qualitative Study of Australian Doctors
Source: Eval Health Prof. 2025 Mar 20;49(1):75–85. doi: 10.1177/01632787251324662 (PMC12852782; doi:10.1177/01632787251324662)
Supplement: Supplemental Material - A Multidisciplinary Model for the Governance of Clinical Innovation: Insights From a Qualitative Study of Australian Doctors [file sj-pdf-1-ehp-10.1177_01632787251324662.pdf]

Exploring innovation: what motivates doctors to use innovative treatments?

In-depth interviews of doctors, regulators, policy makers, health service managers and patient advocates.

Interview topics

This phase of the project consists of semi-structured interviews with individuals who have indicated they are willing to participate in a interview. The purpose of these interviews is to explore stakeholders' views towards clinical innovation and the influences driving doctors to innovate in clinical practice.

This interview guide outlines the topics that will be covered in the interviews, although the specific questions and areas of focus will be adapted and refined as interviews are conducted and specific themes emerge.

Topics for doctors

Project overview
Before we commence the interview, I'd like to give you a brief overview of my PhD project at The University of Sydney.

I am investigating clinical innovation—i.e. innovation that occurs in the course of clinical practice. This includes doctors developing novel solutions to patient problems, or using novel treatments that differ from standard practice. Examples of clinical innovation include prescribing a drug off-label, using a novel stem cell therapy for an indication for which it is not approved, or using a medical device in a different manner than originally intended.

I am particularly interested in exploring the interests that motivate doctors to engage in clinical innovation. An interest in providing high quality patient care is generally assumed to drive doctors' use of innovative treatments. But what of other interests — such as curiosity, the desire to improve treatment options for future patients, or to be the first to master a novel technique? Do these and other interests play a significant role? I am also interested in the barriers that may deter doctors from using or developing innovative treatments—are doctors free to innovate or are there obstacles that may impede their use of innovative interventions in clinical practice?

Any questions?

SECTION ONE: Background information

So to start with, can you tell me briefly about your professional background, and how you came to work in…

SECTION TWO: Interview questions

We will now move to the in depth interview questions.

ONE: Understanding and defining clinical innovation

a. How do you define innovation that occurs in clinical practice?
b. What do you consider to be the defining features of innovation?
c. How do you identify when you, or your colleagues are engaging in clinical innovation?
d. Do you find it easy or difficult to identify when clinical innovation occurs in your area of practice?

TWO: Experience using innovative treatments

a. Do you regularly use innovative treatments in your practice? If so, why? If not, why not?
b. Can you describe an experience where you have used an innovative treatment?
    i. Can you explain what made that treatment "innovative"?
    ii. Were there any procedures/protocols that you had to go through to get approval to use an innovative treatment?
    (Prompt re oversight)
    iii. Can you describe the above procedures/protocols?
    iv. What were your views towards the acceptability of these procedures?
    v. Do you think that additional oversight of clinical innovation is needed in your area of practice? If so, why? If not, why not?

THREE: "Successful" versus "unsuccessful" clinical innovation

a. Can you give me an example of a successful clinical innovation in your area of practice? (For example, one that improved patient outcomes significantly, or reduced time or costs).
b. Can you give me an example of when a clinical innovation went badly in your area of practice? (For example, one that harmed patients, or was later discovered to be ineffective).

(Prompt: we will return to this later in the interview)

FOUR: The interests driving doctors' decisions to use (or avoid using) an innovative treatment

a. Why did you use an innovative treatment as opposed to an existing treatment option?
b. What interests influenced your decision to use an innovative treatment?

(Prompts: for example, an interest in ensuring your patient receives the best possible care, or an interest in alleviating suffering, or an interest in trying a new intervention, etc)

c. Were there any external factors that influence your decision?

(Prompts: for example, a lack of alternate options or pressure from patients, lack of approval from institute, lack of funding, etc)

d. Were there any factors that made you hesitant about using an innovative treatment?

FIVE: Views and attitudes towards the interests driving doctors use of innovative treatments

a. What do you think are the main reasons why doctors use innovative treatments in general?
b. What interests do you think drive doctors' use of innovative treatments?

(Prompts: do you think an interest in financial gain, or status and reputation may drive some doctors to use innovative treatments?)

c. Thinking back to the unsuccessful innovation you mentioned earlier—do you think that doctors' interests were involved somehow? If so, how? If not, why not?
d. Do you think that certain interests are problematic?
e. Do certain interests require oversight and/or management? If so, what form could this oversight take?

Thank you so much for your time.
